# Efficacy of Live-Attenuated H9N2 Influenza Vaccine Candidates Containing NS1 Truncations against H9N2 Avian Influenza Viruses

**DOI:** 10.3389/fmicb.2017.01086

**Published:** 2017-06-14

**Authors:** Sujuan Chen, Yinbiao Zhu, Da Yang, Yang Yang, Shaohua Shi, Tao Qin, Daxin Peng, Xiufan Liu

**Affiliations:** ^1^College of Veterinary Medicine, Yangzhou UniversityYangzhou, China; ^2^Jiangsu Research Center of Engineering and Technology for Prevention and Control of Poultry DiseaseYangzhou, China; ^3^Jiangsu Co-innovation Center for the Prevention and Control of Important Animal Infectious Disease and ZoonosesYangzhou, China; ^4^Yangzhou Vac Biological Engineering Co., Ltd.Yangzhou, China

**Keywords:** H9N2, avian influenza virus, attenuation, NS1, vaccine

## Abstract

H9N2 avian influenza virus is a zoonotic agent with a broad host range that can contribute genetic information to H5 or H7N9 subtype viruses, which are significant threats to both humans and birds. Thus, there is a great need for a vaccine to control H9N2 avian influenza. Three mutant viruses of an H9N2 virus A/chicken/Taixing/10/2010 (rTX-NS1-73, rTX-NS1-100, and rTX-NS1-128) were constructed with different NS1 gene truncations and confirmed by western blot analysis. The genetic stability, pathogenicity, transmissibility, and host immune responses toward these mutants were evaluated. The mutant virus rTX-NS1-128 exhibited the most attenuated phenotype and lost transmissibility. The expression levels of interleukin 12 in the nasal and tracheal tissues from chickens immunized with rTX-NS1-128 were significantly upregulated on day 3 post-immunization and the IgA and IgG antibody levels were significantly increased on days 7, 14, and 21 post-immunization when compared to chickens that received an inactivated vaccine. rTX-NS1-128 also protected chickens from challenge by homologous and heterologous H9N2 avian influenza viruses. The results indicate that rTX-NS1-128 can be used as a potential live-attenuated vaccine against H9N2 avian influenza.

## Introduction

H9N2 avian influenza viruses (AIVs) are endemic to Chinese poultry farms and cause economic losses to the breeding industry. H9N2 AIVs can also cross species barriers to cause non-lethal human infections (Peiris et al., 1999; Butt et al., 2005). Moreover, H9N2 AIVs can contribute their internal genes to new emerging reassortant viruses such as H7N9 (Wu et al., 2013) and H10N8 (Chen et al., 2014), which are great threats to public health. Thus, effective prevention and control of H9N2 avian influenza is crucial.

Vaccination is the most effective method of prophylaxis against influenza. Currently in Mainland China, only inactivated vaccines are licensed for use to control H9N2 subtype AIVs. However, recently there were reports that H9N2 subtype avian influenza still causes outbreaks on chicken farms for which H9N2 AIV immunization had been implemented (Liu et al., 2003; Li et al., 2005). The failure of vaccination based on inactivated vaccine can be due in part to the antigenic drift of H9N2 (Sun et al., 2010).

Unlike inactivated vaccines that only induce humoral immune responses, live-attenuated vaccines (LAVs) can stimulate humoral, cellular and mucosal immune responses, which provide greater cross-protection and longer-lasting immunity (Suguitan et al., 2006; Joseph et al., 2008). Furthermore, LAVs can also be administered using aerosols or liquids (Allan et al., 1978). Cold-adapted live-attenuated influenza viruses generated through consistent low-temperature cultivation have exhibited good efficacy and safety in human populations (Fleming et al., 2006). Besides cold-adapted LAVs, some influenza mutant viruses of subtypes H1 (Zhou et al., 2010), H5 (Talon et al., 2000), and H7 (Wang et al., 2008) expressing truncated NS1 genes display highly attenuated phenotypes both *in vivo* and *in vitro*, with potential to become live-attenuated vaccine candidate strains.

Given that H9N2 subtype AIVs contribute gene segments to novel reassortant viruses such as H7N9 (Wu et al., 2013) and H10N8 (Chen et al., 2014), as well as their own potential threat to public health, it is crucial to develop an effective live-attenuated vaccine to control H9N2 avian influenza. Wei et al. (2016) show that a cold-adapted live-attenuated H9N2 subtype AIV vaccine can provide protection of chickens from homologous and heterogenous H9N2 viruses. However, construction of H9N2 subtype AIV with truncated NS1 gene as a live vaccine for poultry has not been reported. Here we constructed three NS mutant viruses (rTX-NS1-73, rTX-NS1-100, and rTX-NS1-128) of H9N2 subtype AIV by using reverse genetics. These mutant viruses were attenuated to different degrees in chickens. The virus rTX-NS1-128 was most attenuated, induced humoral, cellular, and mucosal immune response, and protected chickens from challenge by homologous and heterologous wild-type H9N2 viruses.

## Materials and Methods

### Biosafety and Animal Care

All experiments were conducted in BSL-2 under the guidelines of Jiangsu Laboratory Animal Welfare and Ethical of Jiangsu Administrative Committee of Laboratory Animals (Permit number SYXKSU-2007-0005).

### Viruses and Cells

H9N2 AIVs A/chicken/Shanghai/F/98 (F98) and A/chicken/ Taixing/10/2010 (TX) (Zhu et al., 2015a,b) were propagated in 10-day-old specific-pathogen-free (SPF) embryonated chicken eggs. The allantoic fluids containing virus were harvested and stored at -70°C. Human embryonic kidney cells (293T) and Madin-Darby canine kidney (MDCK) cells were cultured in Dulbecco’s modified Eagles’ medium (DMEM) with 10% (v/v) fetal bovine serum (HyClone, Logan, UT, United States) at 37°C and 5% CO_2_.

### Generation and Propagation of Mutant Viruses

**FIGURE 1:**
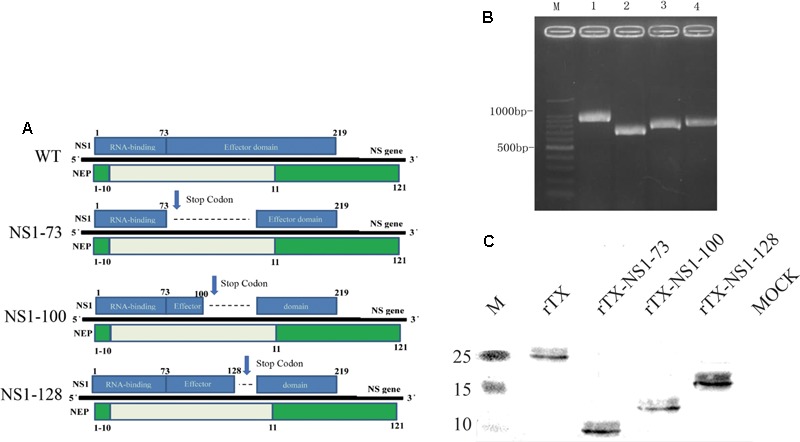
Generation of NS1 mutant viruses. **(A)** Schematic of the NS segment from WT H9N2 AIV and the NS mutations. NS1 is translated from the full-length mRNA and is shown on the top of the gene; NEP is translated from spliced mRNA and is shown below the gene. Selected amino acid positions are labeled for NS1 and NEP. NS1-73, NS1-100, NS1-128 express truncated NS1 but intact NEP. **(B)** NS amplification of the WT and NS mutant viruses. Viral genomes were amplified using RT-PCR and the length of the NS vRNA amplicons was examined using agarose gel electrophoresis. Lane 1, 100 bp marker; Lane 2, WT; Lane 3, NS1-73; Lane 4, NS1-100; Lane 5, NS1-128. **(C)** Western blot analyses. Whole cell lysates obtained from MDCK cells infected with the mutant and wild-type viruses at an MOI of 1 for 3 h were subjected to SDS-PAGE and western blot analysis. Protein bands were visualized using enhanced chemiluminescence.

Wild-type (WT) H9N2 AIV TX was created by plasmid-based reverse genetic technology (Hoffmann et al., 2000). The cDNAs of the eight gene segments of TX were cloned into plasmid pHW2000, confirmed by DNA sequencing, and named as pHW291-PB2, pHW292-PB1, pHW293-PA, pHW294-HA, pHW295-NP, pHW296-NA, pHW297-M, pHW298-NS. Deletions were introduced into the plasmid pHW298-NS using overlap-PCR to generate the three mutant NS segments (**Figure 1A**): pHW298-NS 1-73, pHW298-NS 1-100, and pHW298-NS 1-128. Nucleotides 220-456 (NS1 GenBank Accession Number: JN653684) were replaced by stop codons to generate pHW298-rTX-NS1-73; nucleotides 301–456 were replaced by stop codons to generate pHW298-NS 1-100; nucleotides 385–456 were replaced by stop codons to generate pHW298-NS 1-128. The recombinant viruses rTX, rTX-NS1-73, rTX-NS1-100, and rTX-NS1-128 were generated by co-transfection of eight reverse-genetic plasmids with or without the substitution plasmids pHW298-NS into co-cultured 293T/MDCK cells. Plasmids (300 ng) encoding each gene segment were mixed and incubated with 15 μl of Lipofectamine 2000 (Invitrogen) at room temperature for 15 min. The Lipofectamine-DNA mixture was transferred to 80% confluent 293T/MDCK cells co-cultured in 35 mm dishes and incubated at 37°C with 5% CO_2_ for 8 h. Transfection supernatants were replaced with 2 ml of Opti-MEM 1 medium (Invitrogen) plus 0.3% BSA fraction V (Invitrogen) and 2 μg/ml TPCK-trypsin (Worthington, Lakewood, NJ, United States). Three days post-transfection, the supernatants were collected and subsequently inoculated into 7-day-old embryonated chicken eggs with incomplete Interferon (IFN)-system response for virus propagation (P1 stock). Viral stocks were then inoculated into 10-day-old SPF embryonated chicken eggs to obtain viral stocks. Viral stocks were aliquoted and subsequently stored at -70°C for additional experiments. Viral titers were determined by 50% tissue culture infectious dose (TCID50) in MDCK cells. Sequencing was performed to confirm the identity of the NS gene.

### Western Blot Analysis

Confluent MDCK cells were infected with the mutant viruses and WT virus at a multiplicity of infection (MOI) of 1 for 1 h at 37°C in 5% CO_2_. The infected cells were washed three times with PBS and then fresh opti-MEM (Thermo Fisher Scientific) with 2 μg/ml TPCK trypsin was added. Viral proteins extracted from cells at 3 h post-infection were electrophoresed on a 15% Tris–glycine gel and transferred onto a PVDF membrane. The membrane was blocked in 5% fat-free milk and incubated with polyclonal antibody (anti-NS_1_ of H9N2 AIV mouse serum), followed by incubation with horseradish peroxidase (HRP)-conjugated goat anti-mouse antibodies (EMD Chemicals Inc., San Diego, CA, United States). Protein bands were visualized using enhanced chemiluminescence (Thermo Fisher Scientific Inc., Rockford, IL, United States) on a chemiluminescence imaging analysis system.

### Replication Kinetics in Cell Cultures and SPF Embryonated Eggs

Confluent monolayers of MDCK cells were infected with rTX or NS mutant viruses at a MOI of 0.001 in triplicate. One-hour post-infection (hpi), the inoculum was removed, and cells were washed twice and added with DMEM supplemented with 0.15% BSA fraction V and 2 μg/ml TPCK-trypsin. Supernatants were collected at 12, 24, 48, and 72 hpi and titrated in MDCK cells.

Ten-day-old SPF embryonated chicken eggs were infected with each virus (diluted 10-fold in PBS). After 72 h of incubation at 37°C, allantoic fluids were harvested and used in HA assays to calculate the EID_50_ according to the method of Reed and Muench (1938).

### Pathogenicity in Chickens

The virulence of the viruses was determined in 4-week-old SPF chickens. Eleven chickens were intranasally infected with 10^6^ EID_50_/0.2 ml of rTX or each NS mutant virus and three infected chickens were sacrificed on 3 and 5 days post-inoculation (dpi) to collect trachea and lung tissues for virus titration. In addition, viral shedding was also determined by titrating oropharyngeal and cloacal swabs collected and suspended in 1 ml PBS on both 3 and 5 dpi. All tissue and swab samples were titrated for viral infectivity by EID_50_.

### Dose-Response and Transmissibility Study in Chickens

Ten groups of five 4-week-old SPF chickens were intranasally inoculated with 10^3^–10^7^ EID_50_/0.2 ml of rTX or rTX- NS1-128. Five additional chickens were added to each group at 24 h post-challenge for contact transmission studies. Oropharyngeal and cloacal swabs were collected on both 3 and 5 dpi for viral shedding determinations. Blood was collected to isolate serum on 21 dpi for seroconversion assays.

### Cytokine mRNA Expression and IgA and IgG Secretion in Mucosa

Groups of twenty five 4-week-old SPF chickens were inoculated intranasally with 10^6^ EID_50_ of rTX-NS1-128 or PBS in 0.2 ml, or inoculated subcutaneously with 0.2 ml oil-emulsion inactivated vaccine of 10^6^ EID_50_ rTX. Chickens were euthanized on days 1, 3, 7, 14, and 21 post-immunization. Tracheal and nasal samples of inoculated chickens on days 1 and 3 post-immunization were collected for RNA extraction. The relative expression levels of IL-2, IL-6, and IL-12 were determined using real-time RT-PCR with the following primers: IL-2 (Forward:5′-GAACCTCAAGAGTCTTACGGGTCTA-3′; Reverse: 5′-ACAAAGTTGGTCAGTTCATGGAGA-3′), IL-6 (Forward: 5′-CGGCAGATGGTGATAAATCC-3′; Reverse: 5′-CCCTCACGGTCTTCTCCATA-3′), IL-12 (Forward: 5′-ACCAGCCGACTGAGATGTTC-3′; Reverse: 5′-GTGCTCCAGGTCTTGGGATA-3′), and internal reference gene β-actin (Forward: 5′-ATGAAGCCCAGAGCAAAAGA-3′; Reverse: 5′-GGGGTGTTGAAGGTCTCAAA-3′). Lavage liquids from nasal cavity and trachea of inoculated chickens were collected at 7, 14, and 21 days post-immunization by repeated washing with 0.5 ml of PBS. The lavage liquids were centrifuged at 3,000 × *g* at 4°C for 15 min. The supernatant was collected for detection of AIV specific secretory IgA (sIgA) and IgG using ELISA. The coating antigen was purified rTX (10 μg/ml), the lavage liquids were used as test samples, and goat anti-chicken IgA or IgG antibody (1:10,000 diluted in PBS; Bethyl Laboratories, Inc.) was used as second antibody, followed by HRP-linked rabbit anti-goat IgG (1:20000 diluted in PBS; Medical & Biological Laboratories Co., Ltd.). The absorbance was read at 450 nm using a microplate reader.

### Vaccination and Challenge in Chickens

Four-week-old SPF chickens were inoculated intranasally with 10^6^ EID_50_ virus or PBS in 0.2 ml (*n* = 10 per group). Three weeks after inoculation, chickens were challenged intranasally with 10^6^ EID_50_ of H9 subtype viruses (TX or F98) to determine protective efficacy. Chickens were monitored daily for morbidity and mortality after challenge, and viral shedding in the swabs was evaluated at days 3 and 5 post-challenge.

### Statistical Analysis

Comparisons of experimental groups were estimated using *t*-tests with two-tailed analysis to determine significant differences. *p*-values < 0.05 were considered statistically significant.

## Results

### Mutant Virus Rescue and Western Blotting Analyses

Three different NS1 gene truncations encoding either 73, 100, or 128 amino acids were constructed in the backbone of H9N2 influenza virus (TX) using reverse genetics. The rescued recombinant viruses were confirmed by RT-PCR amplification (**Figure 1B**) and sequence analysis of NS gene (**Supplementary Figure S1**). The three NS1 mutants were purified using limited-dilution method through 10-day-old embryonated chicken eggs. After purification, the variants were passaged at least five times in 10-day-old embryonated chicken eggs. No additional mutations other than the engineered NS1 truncations were observed.

To analyze expression of truncated viral NS1 protein, western blot was performed to examine the mobility change of the NS1 protein. The NS1 proteins from rTX-NS1-73, rTX-NS1-100, and rTX-NS1-128 viruses showed a reduced molecular weight as expected, when compared to the rTX virus (**Figure 1C**), indicating successful construction of the mutant viruses.

### Characterization of Mutant Viruses

The growth properties of the NS1 mutant viruses and wild type virus rTX were compared in MDCK cells and 10-day-old SPF embryonated chicken eggs. All viruses replicated well in SPF embryonated chicken eggs with their titers ranging between 7.47 and 8.08 EID_50_/0.1 ml. Although the titer of each mutant virus was lower than that of wild type virus, there was no significant difference among them (**Table 1**).

**Table 1:** EID_50_ of WT and NS-truncated viruses.

Virus	Log_10_EID_*50*_/0.1 ml
rTX	8.08
rTX-NS1-73	7.47
rTX-NS1-100	7.66
rTX-NS1-128	7.65

**FIGURE 2:**
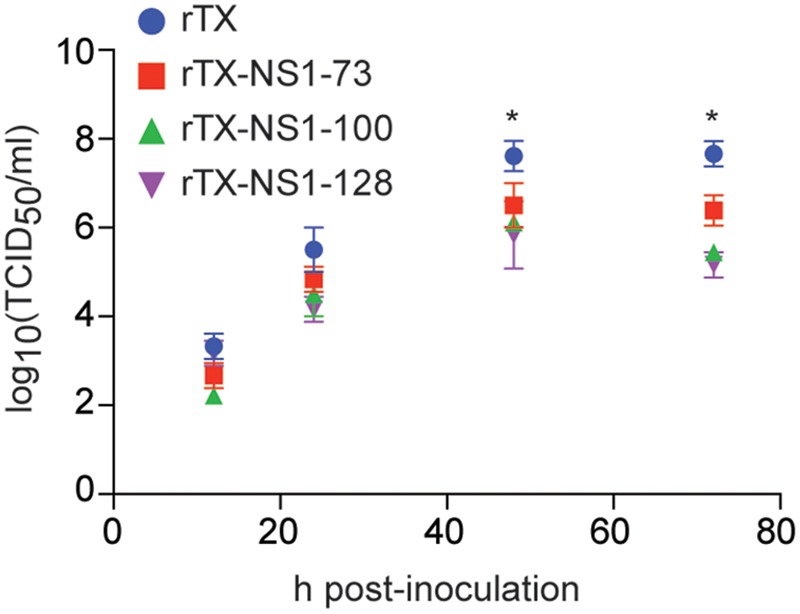
Virus replication in MDCK cells. Cells were infected at a multiplicity of infection of 0.001. Viruses at indicated times post-inoculation were titrated in MDCK cells and expressed as log10 TCID_50_/ml. The mean and standard errors are shown from three independent experiments. Asterisks represent *p*-values < 0.05.

All mutant viruses grew similarly in MDCK cells and their titers peaked at 48 h (between 6.02 and 6.50 TICD_50_/ml) post-infection (**Figure 2**). The titers of each mutant virus were significantly lower than that of wild type virus at 48 and 72 hpi. Neither the wild type rTX nor the mutant viruses replicated in Vero cells.

### Attenuation of Mutant Viruses in SPF Chickens

To examine the level of attenuation of the NS1 mutant viruses, chickens were inoculated intranasally with 10^6^ EID_50_ of the wild-type and mutant viruses. No experimentally infected chicken had clinical signs of disease after 14 dpi.

**FIGURE 3:**
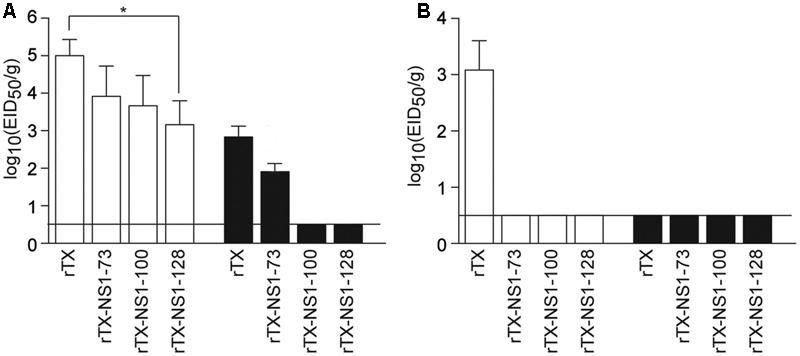
Pathogenicity in chickens. **(A)** Replication of WT and NS mutant viruses in chicken tracheal tissues. **(B)** Replication of WT and NS mutant viruses in chicken lung tissues. Chickens were intranasally infected with 10^6^ EID_50_/0.2 ml of rTX or each NS mutant virus; three infected chickens were sacrificed on 3 (open bars) and 5 (closed bars) days post-inoculation (dpi) to collect tissue samples. All tissue samples were titrated for viral infectivity using EID_50_ assays. The mean and standard errors are shown. Asterisk represent *p*-values < 0.05. Horizontal bar indicates the limit of detection.

In trachea tissues on 3 dpi, the titer of rTX (5.0±0.43 EID_50_/g) was higher than that of rTX-NS1-73 (3.75 ± 0.66 EID_50_/g), rTX-NS1-100 (3.66 ± 0.80 EID_50_/g), and rTX-NS1-128 (3.16 ± 0.64 EID_50_/g), with significant difference between rTX and rTX-NS1-128 (*p* < 0.05). On 5 dpi, only rTX and rTX-NS1-73 were able to replicate in trachea tissues, at titers of 2.83 ± 0.29 and 1.74 ± 0.20 EID_50_/g, respectively (**Figure 3A**). In lung tissues, only wild type rTX was able to replicate on 3 dpi. The mutant viruses did not replicate in lungs at 3 dpi. No viruses were detected in 5 dpi samples (**Figure 3B**).

Viral shedding level of mutant viruses and wild type virus were assayed through titrating oropharyngeal and cloacal swabs (**Table 2**). On 3 dpi, rTX, rTX-NS1-73, rTX-NS1-100, and rTX-NS1-128 viruses were all shed from oropharynx samples are viral shedding rates of 100, 90, 90, and 80%, respectively. On 5 dpi, rTX was still shed from all samples while the shedding rates of rTX-NS1-73 and rTX-NS1-100, and rTX-NS1-128 were reduced to 80, 70, and 50%, respectively. On 7 dpi, only rTX was detected in oropharyngeal swabs. No NS1 mutant virus was detected in cloacal swabs, while rTX was detectable in cloacal swabs on both 3 and 5 dpi. The titer of rTX in oropharyngeal swabs (6.05 ± 0.55 EID_50_/0.1 ml) was significantly higher than the titers of each NS1 mutant virus on 3 dpi (**Table 2**). HI antibody titers induced by NS1 mutant viruses were similar to that induced by rTX at 21 dpi (**Table 2**).

**Table 2:** Viral shedding and titration in chicken oropharyngeal and cloacal swabs.

	3 dpi		5 dpi		7 dpi		HI
Virus	O^a^	C^b^	O	C	O	C	(log2)^c^
rTX	10/10^d^	10/10	10/10	8/10	2/10	0/10	10
	(6.05 ± 0.55^e^)		(2.13 ± 0.55)		(1.16 ± 0.28)		
rTX-NS1-73	9/10	0/10	8/10	0/10		0/10	10
	(3.55 ± 0.26)		(1.25 ± 0.38)				
rTX-NS1-100	9/10	0/10	7/10	0/10	0/10	0/10	10
	(2.94 ± 0.50)		(1.12 ± 0.39)				
rTX-NS1-128	8/10	0/10	5/10	0/10	0/10	0/10	9
	(1.94 ± 0.27)		(1.04 ± 0.27)				

*^a^Oropharyngeal swab.*

*^b^Cloacal swab.*

*^c^HI titer of chickens at 21 days post-inoculation.*

*^d^# of positive chickens/total chickens used.*

*^e^Virus titers: log10 EID_50_/0.1 ml ± SD.*

### Dose-Response and Transmission Study of rTX-NS1-128 in Chickens

Since rTX-NS1-128 was the most attenuated virus but had similar immunogenicity as compared to the other viruses, it was used in subsequent dose-response and transmission studies to evaluate its potential as a live vaccine. Several groups of chickens were inoculated intranasally with rTX and NS1 mutant viruses at doses ranging from 10^3^ to 10^7^ EID_50_. One day after inoculation, five chickens were housed with virus-inoculated chickens as contact groups. Virus rTX was shed from all oropharynx and cloacal samples from chickens inoculated at 10^5^–10^7^ EID_50_. rTX was also transmitted to contact group chickens. Viral shedding rates decreased to ∼60% when chickens were inoculated with 10^4^ EID_50_ rTX. Inoculating chickens with 10^3^ EID_50_ rTX did not yield virus from oropharynx and cloacal samples from either inoculated or contact groups, but did lead to seroconversion in all chickens on 21 dpi (**Table 3**). In contrast to virus rTX, rTX-NS1-128 was only shed from chickens inoculated with 10^6^ to 10^7^ EID_50_. However, rTX-NS1-128 was not transmitted to contact chickens and did not lead to seroconversion (**Table 3**).

**Table 3:** Transmission and dose-response study of wild-type H9N2 (rTX) and NS1-truncated H9N2 (rTX- NS1-128) viruses in chickens.

		3 dpi		5 dpi		
Virus/Dose (EID_50_)	Transmission method	O^a^	C^b^	O	C	Seroconversion^c^
rTX – 10^7^	Inoculation	5/5^d^	5/5	5/5	5/5	5/5
	Contact	5/5	5/5	5/5	5/5	5/5
rTX – 10^6^	Inoculation	5/5	5/5	5/5	5/5	5/5
	Contact	5/5	5/5	5/5	5/5	5/5
rTX – 10^5^	Inoculation	5/5	5/5	5/5	5/5	5/5
	Contact	5/5	5/5	5/5	5/5	5/5
rTX – 10^4^	Inoculation	3/5	3/5	3/5	2/5	5/5
	Contact	2/5	2/5	2/5	1/5	5/5
rTX – 10^3^	Inoculation	0/5	0/5	0/5	0/5	5/5
	Contact	0/5	0/5	0/5	0/5	5/5
rTX-NS1-128 - 10^7^	Inoculation	5/5^d^	5/5	5/5	5/5	5/5
	Contact	0/5	0/5	0/5	0/5	0/5
rTX-NS1-128 – 10^6^	Inoculation	4/5	0/5	3/5	0/5	5/5
	Contact	0/5	0/5	0/5	0/5	0/5
rTX-NS1-128 – 10^5^	Inoculation	0/5	0/5	0/5	0/5	3/5
	Contact	0/5	0/5	0/5	0/5	0/5
rTX-NS1-128 – 10^4^	Inoculation	0/5	0/5	0/5	0/5	0/5
	Contact	0/5	0/5	0/5	0/5	0/5
rTX-NS1-128 – 10^3^	Inoculation	0/5	0/5	0/5	0/5	0/5
	Contact	0/5	0/5	0/5	0/5	0/5

*^a^Oropharyngeal swab.*

*^b^Cloacal swab.*

*^c^HI titer of chickens 21 days post-inoculation or post-exposure. HI < 4log2 was considered negative.*

*^d^# of positive chickens/total chickens used.*

### Cytokine mRNA Expression and IgA and IgG Secretion in Mucosa

**FIGURE 4:**
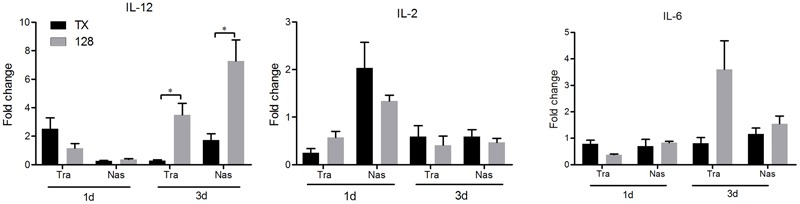
Real-time quantification RT-PCR of cytokine gene expression. Chickens were immunized at 4 weeks of age, and the trachea and nasal tissues were sampled at 1 and 3 dpi. Total RNA was extracted and equal amounts of RNA (1 μg) were used for RT-PCR. Gene expression was normalized to the β-actin gene expression level and presented as the fold increase relative to PBS-treated chickens. Data represent the mean fold changes ± standard errors. Asterisks represent *p*-values < 0.05.

**FIGURE 5:**
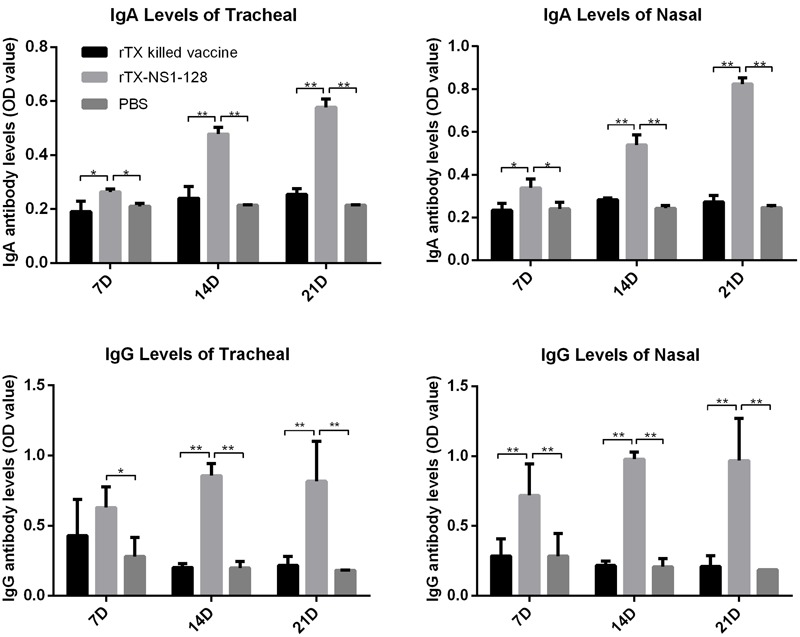
Detection of specific IgA and IgG level in mucosa. Chickens were immunized at 4 weeks of age, and lavage fluids from the trachea and nasal tissues were sampled at 7, 14, and 21 dpi. The antibody levels were detected using ELISA. All data are shown as the mean ± standard errors. Asterisks represent *p*-values < 0.05. Double asterisks represents *p*-values less than 0.01. OD, optical density.

To evaluate aspects of cellular and mucosal immunity, cytokine expression and secretion of IgA and IgG in nasal trachea tissues were determined. IL-12 expression in the tracheal and nasal tissues of rTX-NS-128 immunized chickens was significantly higher than that in killed rTX vaccine immunized chickens 3 days after immunization (*P* < 0.05) (**Figure 4**). There was no significant difference in IL-2 and IL-6 expression between rTX-NS-128 and killed rTX vaccine immunized chickens. Both IgA and IgG levels in the tracheal and nasal lavage fluids induced by rTX-NS1-128 were significantly higher than that induced by killed rTX vaccine at 7, 14, and 21 dpi (**Figure 5**).

### Immune Responses and Protective Efficacy of rTX-NS1-128 in Chickens

**FIGURE 6:**
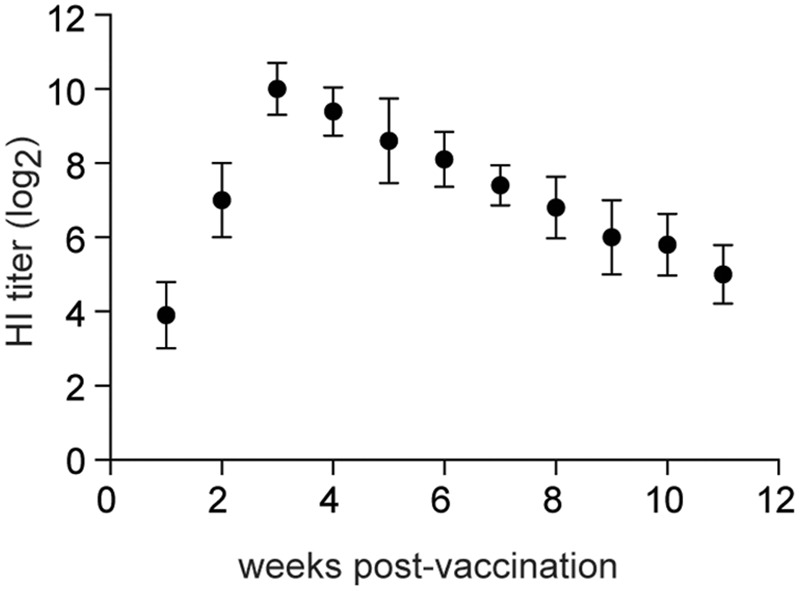
Hemagglutination inhibition titers. Ten chickens were i.n. vaccinated with 1 × 106 log10EID_50_/0.2 ml of rTX-NS1-128 and were bled at 1-week intervals after vaccination. HI titers in sera were determined with wild-type H9N2 TX and 0.5% chicken red blood cells. The tissues of tracheas and lungs were collected and homogenized before log10 EID_50_/ml were determined. The mean and standard errors are shown.

Specific-pathogen-free chickens (4-weeks old) were inoculated intranasally with 10^6^ EID_50_ of rTX-NS1-128. HI titers increased rapidly to 7log2 at 2 weeks post-inoculation (wpi) and peaked to 10log2 at 3 wpi. High HI titers lasted for about 8 weeks and sharply decreased to 5log2 at 11 wpi (**Figure 6**). Four-week-old SPF Chickens were inoculated intranasally with 10^6^ EID_50_ of rTX-NS1-128 and were challenged with homologous (TX) and heterologous (F98) H9N2 viruses on day 21 post-inoculation. No virus was shed from chickens vaccinated with rTX-NS1-128, while chickens in the control group shed both viruses after challenge (**Table 4**). These data suggest that rTX-NS1-128 can provide complete protection in chickens against either H9N2 virus.

**Table 4:** Protective efficacy induced by rTX-NS1-128 in SPF chickens.

		3 dpi		5 dpi		7 dpi	
Group	Challenge virus	O^a^	C^b^	O	C	O	C
rTX-NS1-128	F98 (H9N2)	0/10^c^	0/10	0/10	0/10	0/10	0/10
rTX-NS1-128	TX (H9N2)	0/10	0/10	0/10	0/10	0/10	0/10
Control	F98 (H9N2)	10/10	9/10	10/10	9/10	2/10	0/10
Control	TX (H9N2)	10/10	10/10	10/10	10/10	3/10	2/10

*^a^Oropharyngeal swab.*

*^b^Cloacal swab.*

*^c^# of positive chickens/total chickens used.*

## Discussion

Oil-emulsion inactivated vaccines are widely used in China to control H9N2. However, antigenic drift has resulted in several new H9N2 AIV genotypes (Li et al., 2005; Jiang et al., 2012) and the protective efficacy of existing H9N2 vaccines has decreased (Sun et al., 2010). Live attenuated vaccines have several advantages, such as induction of humoral, mucosal, and cellular immunity, convenient administration, and cross-protective efficacy (Joseph et al., 2008). Cold-adapted vaccine strains such as LAV (Maassab and Bryant, 1999) have provided excellent protective efficacy in mice and chickens (Suguitan et al., 2006). Altering the NS1 gene using reverse genetics is an alternative approach.

The influenza A virus NS1 protein is a multifunctional protein that plays important roles in virus replication and antagonizing host antiviral responses, especially by blocking type I interferon (IFN) responses (Hale et al., 2008). The NS1 protein is 230 amino acids long and contains two distinct functional domains. The N-terminal RNA-binding domain (residues 1–73) prevents the synthesis of IFN-α/β by inhibiting dsRNA-dependent protein kinase R activation. The effector domain (residues 74–230) possesses several different functional epitopes that interact with many different host proteins (Hatada and Fukuda, 1992), including eukaryotic translation initiation factor 4GI (EIF4G) (Aragón et al., 2000), protein kinase R (Li et al., 2006), and cleavage and polyadenylation specific factor (F) (Nemeroff et al., 1998). NS1 is a potent IFN antagonist and mutations/deletions in NS1 result in stronger host stronger IFN responses (Talon et al., 2000; Hale et al., 2008).

Several experimental LAIVs for birds, pigs, and horses have been constructed with NS1 protein truncations (Quinlivan et al., 2005; Vincent et al., 2007; Wang et al., 2008; Hyesun et al., 2016; Shi et al., 2016). Here we generated three NS1 mutant viruses (rTX-NS1-73, rTX-NS1-100, and rTX-NS1-128) by creating C-terminal NS1 truncations. These mutant viruses all replicated in 10-day-old embryonated chicken eggs and MDCK cells but were attenuated relative to the wild-type virus. The degree of attenuation increased as a function of increased NS1 truncation, similar to findings from other studies (Quinlivan et al., 2005).

The nasal mucosa is an effective site for induction of mucosal immunity because of the low threshold for initiation of an immune response. Mucosal immunization also improves local (s-IgA and IgG) antibody responses (Macpherson et al., 2008). We evaluated the potential of the most attenuated mutant virus, rTX-NS1-128, as a live vaccine. The rTX-NS1-128 live vaccine induced enhanced mucosal immunity responses, suggesting that it could more effectively resist infection of H9N2 influenza virus. Cellular immunity in the mucosal site also plays an important role in early immune responses to prevent pathogens from entering the epithelium. Live vaccines can promote the secretion of Th1 cytokines (e.g., IL-2 and IL-12) and these cytokines can further activate natural killer cells to secrete IFN-γ or stimulate T cells to enhance cytotoxic T lymphocytes responses (Min et al., 2001; Rauw et al., 2010). The rTX-NS1-128 live vaccine induced the secretion of Th1 cytokines on the local mucosa at the early stage of immune responses. rTX-NS1-128 stimulated inoculated chickens to produce significant humoral immune responses to the virus. Vaccination with rTX-NS1-128 provided 100% protection to chickens subsequently challenged with homologous (TX) and heterologous (F98) H9N2 AIVs Several studies have showed that the H9N2 inactivated vaccines can only induce humoral immune response and provide complete protection against homologs challenge (Sun et al., 2012; Shin et al., 2016; Wei et al., 2016). Our study demonstrated that the rTX-NS1-128 inducted humoral, cellular and mucosal immune response and provide a cross protection against heterologous challenge, which will be benefit to avoiding failure of immunization by caused antigenic drift variants.

We conclude that rTX-NS1-128 could be used to control and prevent H9N2 AIV infections. Although NS1-truncation-based LAVs appear to be stable (Egorov et al., 1998; Donelan et al., 2003), there is a concern that live-attenuated vaccines may revert to virulence. In the future we will pursue the construction of dual fail-safe LAVs that possess both a truncated NS1 protein as well as temperature-sensitivity.

## Author Contributions

DP, SC, and YZ participated in the design of the study. SC, YZ, DY, YY, and SS performed the experiment. SC, YZ, and TQ analyzed the data and drafted the manuscript. DP and XL planned the experiments and helped write the manuscript. All authors read and approved the final manuscript.

## Conflict of Interest Statement

The authors declare that the research was conducted in the absence of any commercial or financial relationships that could be construed as a potential conflict of interest.

## Supplementary Material

FIGURE S1 Sequence analysis of NS1 gene. Total RNAs were extracted and NS1 genes were amplified for sequence analysis.
