# Supplementary figures and images for: Efficacy of Live-Attenuated H9N2 Influenza Vaccine Candidates Containing NS1 Truncations against H9N2 Avian Influenza Viruses
Source: Front Microbiol. 2017 Jun 14;8:1086. doi: 10.3389/fmicb.2017.01086 (PMC5469905; doi:10.3389/fmicb.2017.01086)

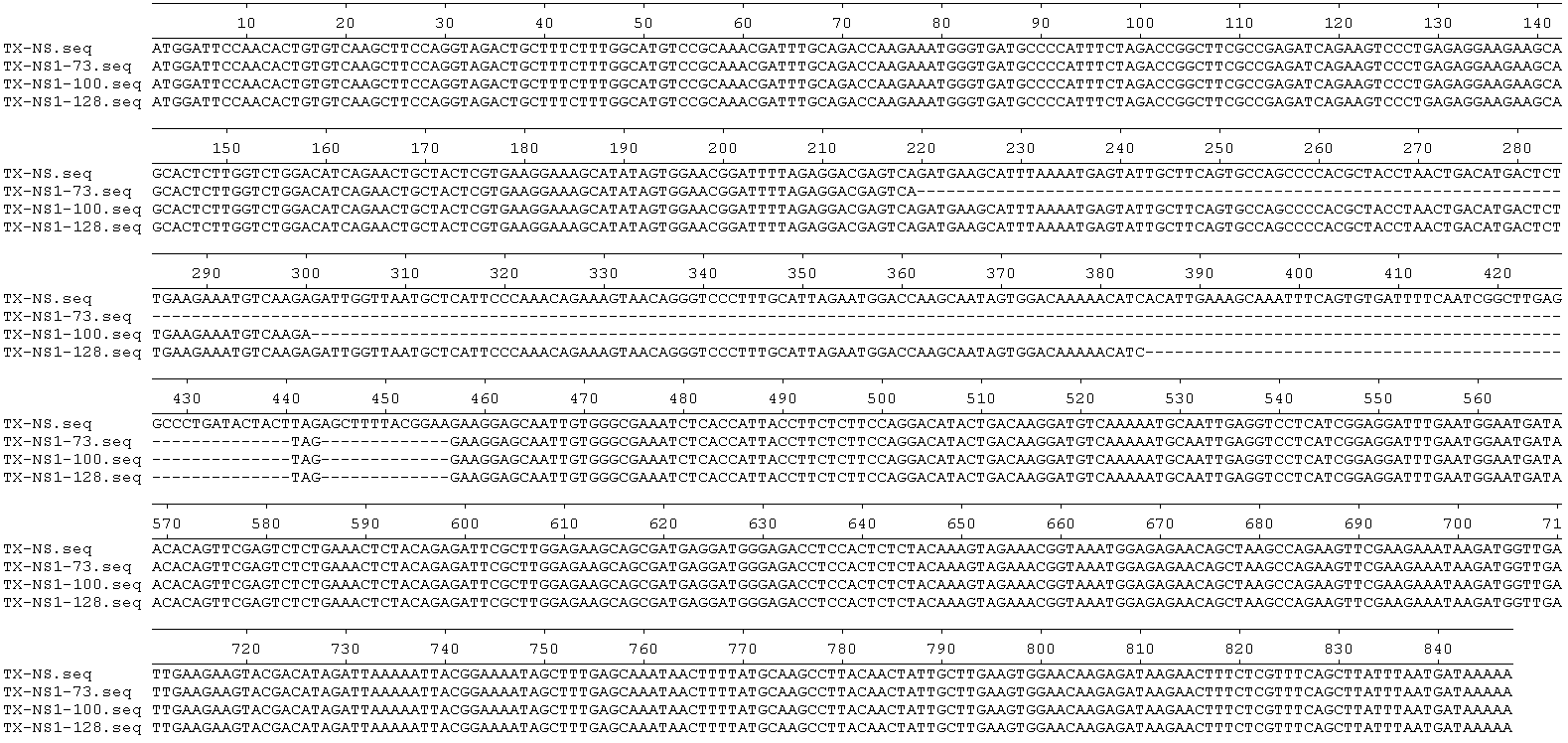

Supplement: FIGURE S1 — Sequence analysis of NS1 gene. Total RNAs were extracted and NS1 genes were amplified for sequence analysis. [file Image_1.TIF]
